# Highly Sensitive Capsaicin Electrochemical Sensor Based on Bimetallic Metal-Organic Framework Nanocage

**DOI:** 10.3389/fchem.2022.822619

**Published:** 2022-02-15

**Authors:** Xiao Fang, Rongshuai Duan

**Affiliations:** Shandong Institute of Commerce and Technology, Jinan, China

**Keywords:** food analysis, capsaicin, electrochemical sensor, MOF, recycled oil, voltammetry

## Abstract

The content of capsaicin can be used as exotic markers of kitchen recycled oil. In this study, a bimetallic MOF nanocage (Fe^III^-HMOF-5) was successfully prepared by a one-step solvothermal method and used for electrode modification to prepare a highly sensitive electrochemical sensor for rapid detection of capsaicin. Capsaicin could be selectively immobilized onto the Fe^III^-HMOF-5 surface during infiltrating adsorption, thus exhibiting very excellent sensing performance. The detection conditions of the sensor were optimized. Under optimum conditions, the electrochemical sensor can linearly detect capsaicin in the range between 1–60 μM with a detection limit of 0.4 μM. In addition, the proposed electrochemical sensor showed excellent stability and selectivity. The real sample tests indicated the proposed electrochemical sensor was comparable to conventional UV spectrophotometry.

## Introduction

Capsaicin-like substances are active ingredients obtained from the fruits of the pepper plant and are characterized by their pungency. Capsaicinoids is a general term for a group of lipophilic alkaloids, including capsaicin, dihydrocapsaicin, hypo-dihydrocapsaicin, homo-dihydrocapsaicin, and hypercapsaicin. Among them, capsaicin and dihydrocapsaicin accounting for more than 90% of lipophilic alkaloids (Chan et al., 2021; Zhao et al., 2021). The application of capsaicin-like substances is very wide. Illegal cooking oil (ICO) is a generic term for all kinds of poor quality oil, among which slop oil from the catering industry is the main raw material of ICO, in addition to frying waste oil, etc. After refining and processing, ICO loses a lot of essential fatty acids, phytosterols and other nutrients, and leaves a lot of fungicides and other harmful ingredients, such as polychlorinated biphenyls (PCBs) and dioxins, which can be very harmful to human health (Granero et al., 2021; H.; Naskar et al., 2021). In 1999 and 2008, crises involving PCBs and dioxin contamination of feed and foodstuffs erupted in Belgium and the Republic of Ireland (Bernard et al., 2002; Jacob et al., 2011), respectively. Capsaicin, as a commonly used flavoring agent, has become an inevitable active ingredient in ICO due to its own lipophilic nature (Sýs et al., 2019; Chiorcea-Paquim et al., 2020; Wu et al., 2022). Therefore, the establishment of a simple, rapid, sensitive and efficient method for the determination of capsaicin cannot only provide technical support for the study of the pharmacological effects and pharmacokinetics of capsaicin, but also the identification of ICO, which is of great significance in the field of maintaining human health and food safety. At present, the commonly used methods for the detection of capsaicin include spectrophotometric method (Hulaswar and Patil, 2019), high performance liquid chromatography (HPLC) (Popelka et al., 2017) and Liquid chromatography-mass spectrometry (GC-MS) (Hamada et al., 2019). The spectrophotometric method is simple and easy to operate, but the anti-interference ability is poor. The HPLC can achieve the effective separation and detection of capsaicin, but the instrumentation is expensive, so it is difficult to promote the use. The GC-MS has good sensitivity and accuracy, however, it has a high detection cost. Therefore, it is necessary to establish a simple, rapid, sensitive and specific method for the determination of capsaicin at low cost.

Electrochemical analysis is a qualitative or quantitative detection technique based on the electrochemical activity of the substance to be measured (Mpanza et al., 2014). Zhong et al. (Zhong et al., 2019) successfully manufactured reduced graphene oxide functionalized with poly dimethyl diallyl ammonium chloride (PDDA) modified palladium nanoparticles (PDDA-rGO/Pd). For sensitive capsaicin determination, they used PDDA-rGO/Pd as the sensing layer. The findings revealed that the nanocomposite had enticing electrocatalytic activity for capsaicin oxidation. This was due to the synergistic action of Pd nanoparticles and graphene nanosheets’ outstanding characteristics. The electrochemical sensor had a dynamic linear range of 320 nM to 64 μM under optimal circumstances, with a detection limit of 100 nM. Temcheon et al. (Temcheon et al., 2019) described a nitrogen-doped mesoporous carbon (N-MC) modified glassy carbon electrode (GCE) with strong electrocatalytic activity for very sensitive capsaicin detection. The electrochemical sensor has a surprisingly low detection limit of 460 nM under ideal conditions, with a wide linear range of capsaicin between 1–10 μM and 10–40 μM. These electrochemical sensors are based on the surface modification of GCE. They have been able to achieve highly sensitive detection of capsaicin, but since GCE is an electrode that needs to be polished, it is suitable for laboratory detection and can hardly be used for field detection. Screen printed carbon electrode (SPCE) has the advantages of simple fabrication, portability and low price (Wang et al., 2017). The modification of the electrode surface by nanomaterials can significantly increase the specific surface area, and the nanomaterials themselves have adsorption and catalytic properties, which can significantly improve the sensitivity of the detection. Metal-organic frameworks (MOFs) are a new type of zeolite-like porous material that has attracted much attention in recent years. It is a general term for a class of polymers with a three-dimensional, periodic mesh structure based on the self-assembly of multiple binding sites of metal nanoparticles, metal clusters or single metal ions with organic ligands using strong interactions such as covalent bonds (Cao et al., 2019; Karimi-Maleh et al., 2021). MOFs not only have a rich topology, but also a high specific surface area and a flexible and versatile pore structure, and have been increasingly used in gas storage, small molecule separation, catalysis, medicine, and other fields in recent years. MOF has a highly repetitive honeycomb pore structure, which increases the specific surface area of electrodes, enhances electrical conductivity and promotes electron transfer (Naghian et al., 2020; Xue et al., 2020). Due to its pore diameter and flexible shape, MOF can specifically bind to the target after grafting or doping with different groups, making it an excellent electrode material for fabrication of electrochemical sensors. In 2008, Riskin et al. (Riskin et al., 2008) started to use MOF in electrochemical sensors. They used a gold electrode as the working electrode and used gold nanoparticles and p-aminothiophenol to form a pore structure with adjustable pores, which conjugated with trinitrotoluene through π-π interaction force. The application of MOFs in electrochemical sensors has developed rapidly in the last decade. Later, Yildiz et al. (Yildiz et al., 2008) improved the above framework to construct a new MOF using gold nanoparticles, quantum dots and p-aminothiophenol. Quantum dots have been used as label for contributing current signal. the In this work, we prepared hollow Fe^III^-HMOF-5 by a one-step solvothermal method. Capsaicin can be concentrated into Fe^III^-HMOF-5 by infiltrating adsorption. Although the electrochemical performance of bare SPCE is not sufficient for highly sensitive detection of capsaicin, the surface modification of Fe^III^-HMOF-5 gives it this potential. The Fe^III^-HMOF-5-modified SPCE becomes a portable electrochemical sensor, which makes it possible to detect capsaicin in the field. In addition, the proposed electrochemical sensor has a wide linear detection range, This which makes the sensor suitable for both ICO and capsaicin detection in peppers.

## Materials and Experiments

### Materials and Instrument

Zinc nitrate hexahydrate, iron acetylacetonate, terephthalic acid, polyvinylpyrrolidone (PVP, Mw = 10,000 g/mol), N, N-dimethylformamide, ethyl acetate and capsaicin were purchased from Aladdin Co. (Shanghai, China). Anhydrous ethanol and methanol were purchased from Sinopharm Group Co. (Shanghai, China). Screen-printed electrode (SPCE) was purchased from Nanjing Youyun Biotechnology Co. (Nanjing, China). The screen-printed carbon electrode consists of an integrated three-electrode system. The working electrode and counter electrode are printed from graphite and the reference electrode is printed from Ag paste containing AgCl. The supporting electrolyte is Britton-Robison (BR) buffer solution. All electrochemical measurements were carried out at a CHI760E working station. The morphology of the prepared Fe^III^-HMOF-5 was observed by transmission electron microscopy (TEM, HT770, Hitachi, Tokyo, Japan). X-ray powder diffraction patterns (XRD) were obtained with an XRD diffractometer (X’pert Pro-1, Panaco, Almelo, The Netherlands). Thermal decomposition was conducted using a thermogravimetric analyzer (Mettler Toledo STARe, Berne, Switzerland). UV-VIS spectra were recorded on Varian CaryScan50 spectrophotometer (VarianInc., Berne, Switzerland). A vacuum centrifugal concentrator (LNG-T96, Huamei Biochemical Instrument Factory, Taicang, China) was used for sample preparation. A Nicolet iS50 spectrometer (Thermo Fisher Scientific, Waltham, MA, United States) was used for collecting the FTIR spectra of samples.

### Preparation of Fe^III^-HMOF-5

Fe^III^-HMOF-5 was synthesized by a one-step solvothermal method (Cheng et al., 2018), in which zinc nitrate hexahydrate and iron acetylacetonate were used as metal nodes, terephthalic acid was used as organic ligand, polyvinylpyrrolidone was used as stabilizer, and N, N-dimethylformamide-ethanol was used as solvent mixture. Zn(NO_3_)·6H_2_O (1200 mg), iron acetylacetonate (1200 mg), terephthalic acid (192 mg), and polyvinylpyrrolidone (4 g) were weighed and dissolved in N, N-dimethylformamide-ethanol mixture (512 ml, v/v = 5:3) with magnetic stirring at room temperature for 10 min. Then, the mixture was transferred into a reactor and heated to 150°C for 6 h. After the reaction, the mixture was cooled to room temperature, centrifuged at 10,000 rpm for 20 min to obtain the precipitate, and washed three times with N,N-dimethylformamide and ethanol, respectively. Finally, the product was dried under vacuum at 40°C for 24 h.

### SPCE Surface Modification and Electrochemical Determination

First, 3 μL of Fe^III^-HMOF-5 aqueous dispersion (500 μg/ml) has been drop-coated on the working electrode of the SPCE and dried naturally (denoted as Fe^III^-HMOF-5/SPCE). Then, the Fe^III^-HMOF-5/SPCE was inserted into an electrochemical cell containing capsaicin. A simple accumulation process is performed before scanning. The accumulation potential was set to −0.1 V and the time was 3 min. Then, differential pulse voltammetry (DPV) was carried out for measurement (potential window: 0.35–0.75 V; pulse amplitude = 50 mV; step amplitude = 4 mV; pulse time = 50 ms; scan rate = 8 mV/s). Cyclic voltammetry (CV) was used for measuring the electrochemical behavior of the electrode and analyte (scan rate = 100 mV/s). The electrodes were reused by dipping the tested SPCE or Fe^III^-HMOF-5/SPCE into ethyl acetate three times to desorb the capsaicin, and then rinsed with water.

### Sample Pre-treatment

The sample pre-treatment method was slightly modified from that reported in the literature (Kachoosangi et al., 2008). Fresh chili peppers were purchased from the local supermarket, ground. 200 mg sample was weighed and added to 12 ml of anhydrous ethanol, sealed and sonicated for 20 min. The sonicated mixture was stirred for 2 h in a magnetic stirrer and then centrifuged at 10,000 rpm for 10 min. Finally, 200 μL of the filtrate was centrifuged to dryness using a vacuum centrifugal concentrator. The sample was re-dissolved with 4 ml of supporting electrolyte solution, and placed in a 20 ml electrochemical cell for measurement. ICO samples were supplied by Department of Quality Standards, Institute of Agricultural Sciences. 5 g of ICO were added into 5 ml methanol and followed by 5 min sonication and 10 min centrifugation at 2000 rpm. The supernatant was filtered through an organic membrane (0.45 μm) and then used for electrochemical analysis (Wang et al., 2020).

## Results and Discussion

### Material Characterizations

**FIGURE 1:**
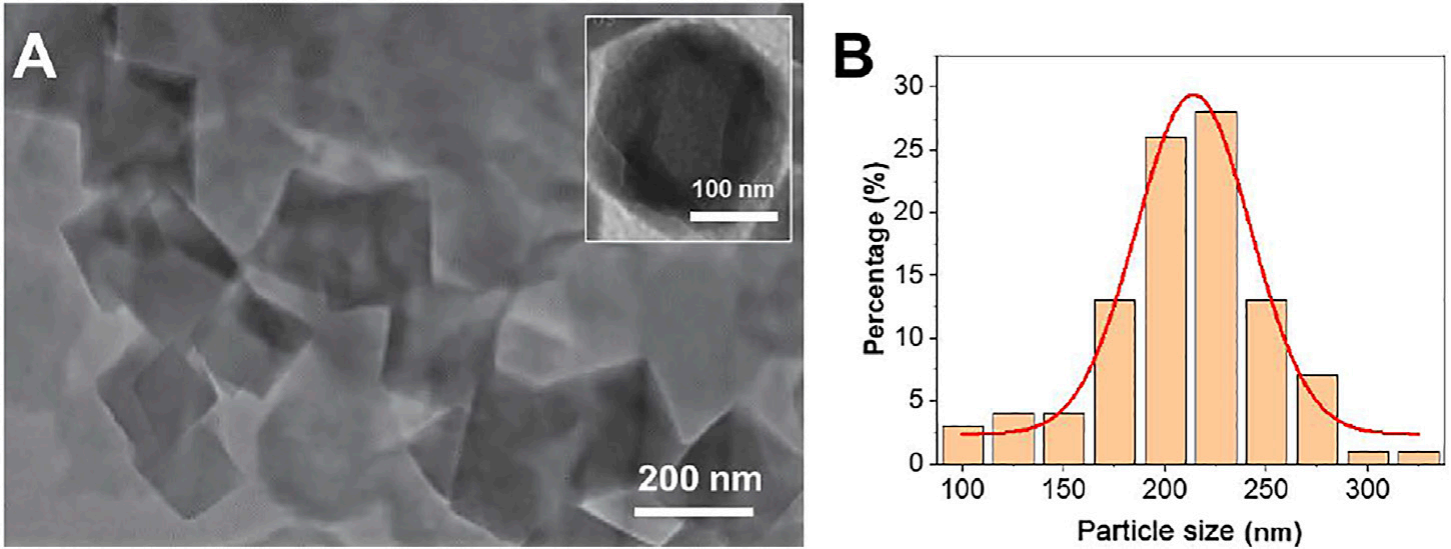
**(A)** TEM image of Fe^III^-HMOF-5. **(B)** Average diameter of the Fe^III^-HMOF-5.

TEM was used to characterize the Fe^III^-HMOF-5 obtained by the one-step hydrothermal method (Figure 1A). It can be seen that Fe^III^-HMOF-5 shows an octahedral structure. The high resolution image (Inset of Figure 1A) shows some fuzzy regions within the octahedra that are cavities in FeIII-HMOF-5. The average diameter is about 211 nm (Figure 1B). The smaller size ensures that the material has a large specific surface area, which not only facilitates the adsorption of capsaicin, but also increases the area for electrochemical reactions.

**FIGURE 2:**
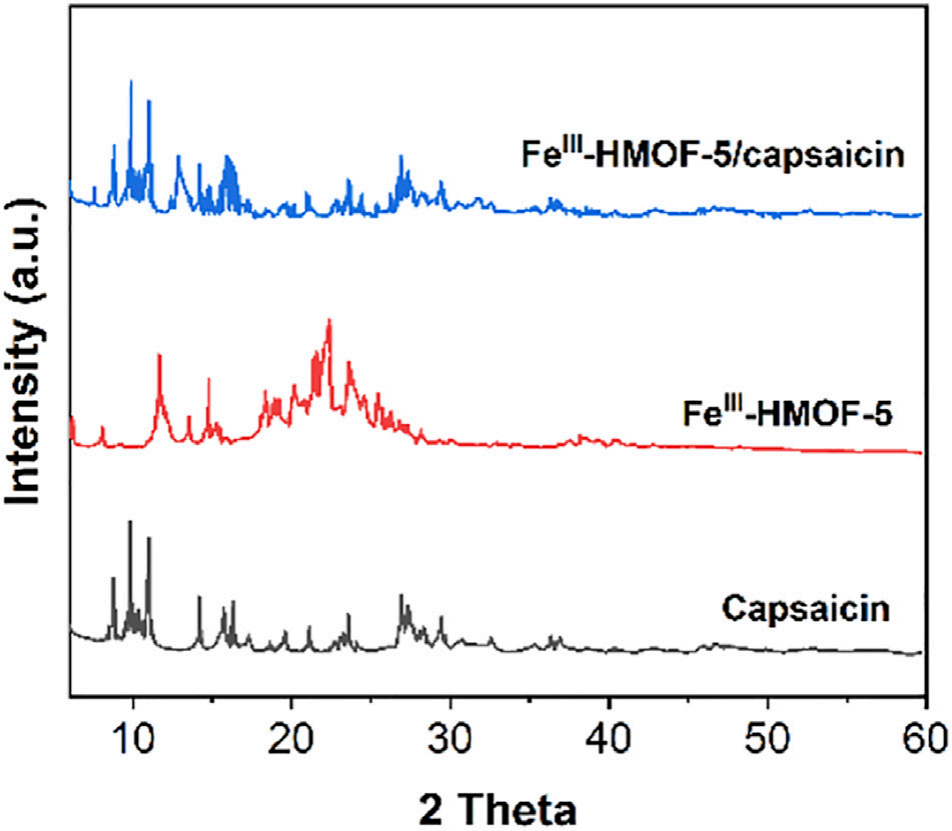
XRD patterns of Fe^III^-HMOF-5, capsaicin and Fe^III^-HMOF-5/capsaicin.

Figure 2 shows the XRD patterns of Fe^III^-HMOF-5, capsaicin and Fe^III^-HMOF-5/capsaicin. It can be seen that both capsaicin and Fe^III^-HMOF-5 show good crystal structures. Faint peaks (19.7°, 18.6°, 20.2°, 24.7°, 25.2°, 26.1°) of Fe^III^-HMOF-5 can be seen after the adsorption of capsaicin by Fe^III^-HMOF-5, suggesting the Fe^III^-HMOF-5 can adsorbed capsaicin into its cavities.

**FIGURE 3:**
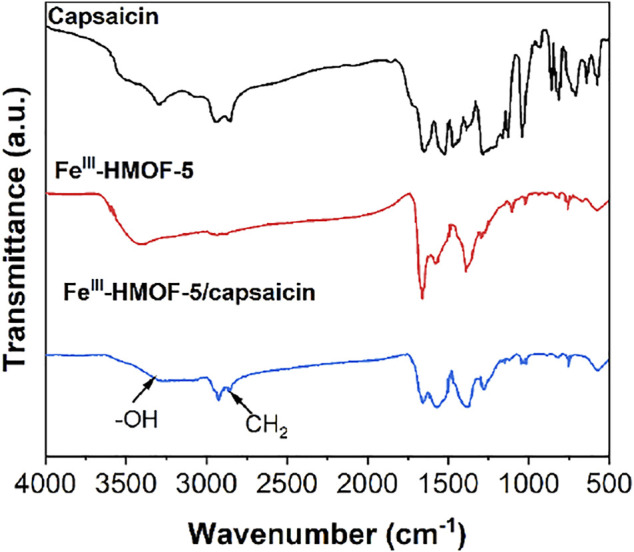
FTIR spectra of capsaicin, Fe^III^-HMOF-5 and Fe^III^-HMOF-5/capsaicin.

We conducted FTIR characterization of the prepared Fe^III^-HMOF-5 and Fe^III^-HMOF-5/capsaicin to determine whether new chemical bonds were formed before and after adsorption. As shown in Figure 3, the spectrum of capsaicin showed a clear peak of CH_2_ at 2,900 cm^−1^ (Kuzminova et al., 2021). The spectrum of Fe^III^-HMOF-5 showed a clear presence of -OH, while the spectrum of Fe^III^-HMOF-5/capsaicin showed a significant weakening of the -OH peak. It can be inferred that the interaction between -NH_2_ of capsaicin and -OH of Fe^III^-HMOF-5 promoted the adsorption of capsaicin (Sharanyakanth and Radhakrishnan, 2020). The more obvious peak of -CH_2_- appeared in the complex, indicating the successful complexation between the Fe^III^-HMOF-5 and capsaicin (Zhao et al., 2020). There is specific adsorption between capsaicin and Fe^III^-HMOF-5 in addition to simple physical adsorption. Supplementary Figure S1 shows the XPS Fe2p results. The peak positions of Fe2p_3/2_ and Fe2p_1/2_ of Fe(III) were blue-shifted from 728.2 eV to 715.3 eV–727.4 eV and 713.7 eV, respectively, after the adsorption of capsaicin by FeIII-HMOF-5, demonstrating that the adsorption of capsaicin was carried out by the interaction with Fe^III^.

**FIGURE 4:**
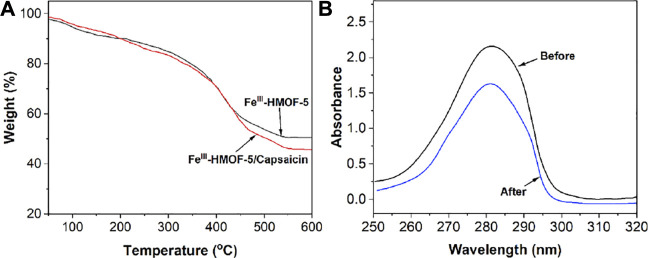
**(A)** TGA curves of Fe^III^-HMOF-5 and Fe^III^-HMOF-5/capsaicin. **(B)** UV-vis spectra of 20 mg/ml of capsaicin before and after adsorption.

TGA was used to study the uptake of capsaicin in Fe^III^-HMOF-5. As shown in Figure 4, the final weights of the TGA curves show that Fe^III^-HMOF-5 possesses a stable metal skeleton, and comparing the differences between the two curves demonstrates that Fe^III^-HMOF-5 can adsorb approximately 5% of its own total amount of capsaicin.

Capsaicin has a clear absorption peak in the UV region, so we also measured the absorption spectrum of capsaicin before and after Fe^III^-HMOF-5 adsorption. As shown in Figure 4B, there was a very significant decrease in the absorption peak of capsaicin at 20 mg/ml after Fe^III^-HMOF-5 absorption.

### Electrode Performance

**FIGURE 5:**
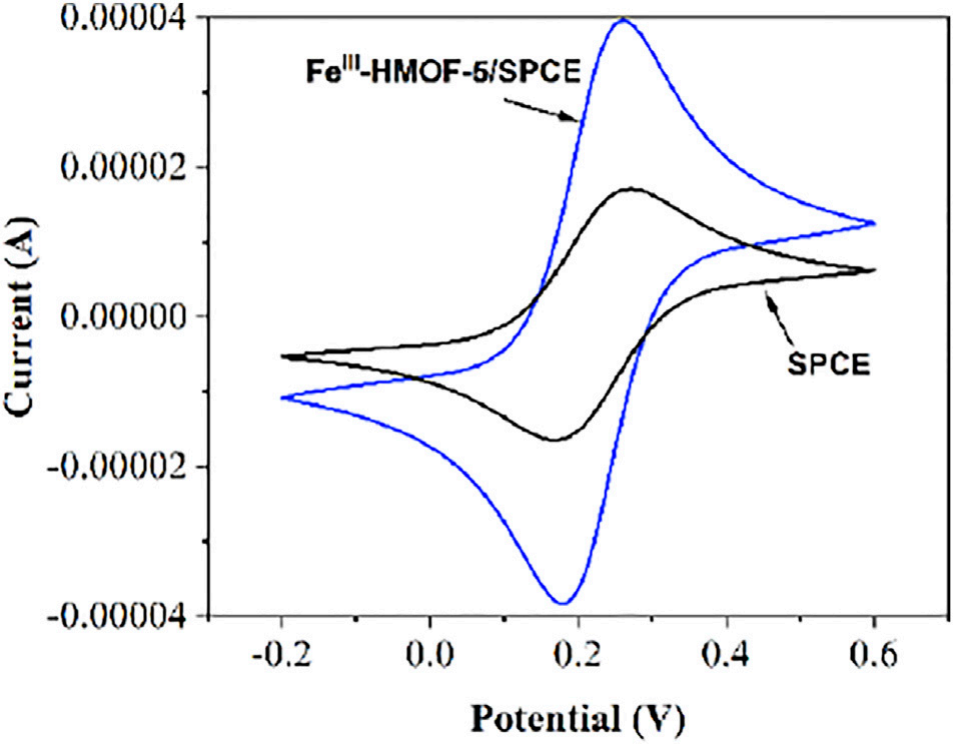
CV curves and of bare SPCE and Fe^III^-HMOF-5/SPCE at 1 mM K_3_[Fe(CN)_6_].

In this experiment, CV was selected for the electrochemical characterization of the modification process. K_3_[Fe(CN)_6_] is widely used probe for characterizing the surface property of the electrode. As shown in Figure 5A, the detection process was carried out in 1 mM K_3_[Fe(CN)_6_]. It can be seen that the bare SPEC produces a pair of obvious redox peaks in the voltage range of −0.2–0.6 V. The peak current value has increased when the SPCE modified with the Fe^III^-HMOF-5, which indicates the large specific surface area and excellent electrical conductivity of Fe^III^-HMOF-5.

### Sensing Optimization and Performance

**FIGURE 6:**
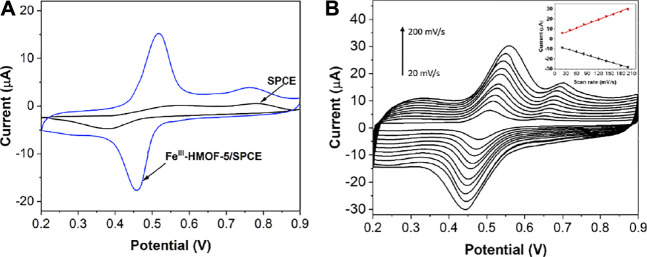
**(A)** CVs of bare SPCE and Fe^III^-HMOF-5/SPCE toward 50 μM capsaicin (pH 1.5; scan rate: 100 mV/s). **(B)** CVs of Fe^III^-HMOF-5/SPCE toward 50 μM capsaicin using scan rate from 20 to 200 mV/s.

Figure 6A shows the CV curves of SPCE and Fe^III^-HMOF-5/SPCE for 50 μM capsaicin. Since capsaicin exhibits excellent electrochemical behavior in an acidic environment, pH 1.5 BR was chosen as the buffer solution. It can be seen that SPCE has a pair of weak oxidation peaks at potentials 0.57 and 0.38 V, indicating that the electrochemical reaction of the untreated electrode on capsaicin is weak. In contrast, the modification of Fe^III^-HMOF-5 significantly improved the response intensity of capsaicin on the electrode surface, with a pair of well-defined sharp redox peaks at potentials 0.53 V, 0.46 V. Capsaicin is known to undergo redox reactions and can thus be identified using electrochemical methods (Ya et al., 2012). The peak at 0.46 V corresponded to the two-electron reduction of the o-benzoquinone (produced by the hydrolysis of the 2-methoxy group) to catechol, whereas peak at 0.53 V corresponded to the reverse process, the two-electron oxidation reaction of catechol to o-benzoquinone (Supplementary Figure S2). Compared with the bare SPCE, the oxidation peak potential of capsaicin was negatively shifted with a positive shift of reduction peak. In addition, the peak potential difference was reduced along with current enhancement indicating that the reversibility of capsaicin on Fe^III^-HMOF-5/SPCE was better. The enhancement of Fe^III^-HMOF-5 towards capsaicin is due to the increase of the specific surface area and active sites of the electrode. On the other hand, the specific binding of Fe^III^-HMOF-5 and capsaicin increased the local concentration. Therefore, it showed a better electrochemical response on capsaicin. Figure 6B shows the CV profiles of Fe^III^-HMOF-5/SPCE towards 50 μM capsaicin with different scan rates. The redox peak current of capsaicin increases with the increase of the scan rate. The oxidation peak potential gradually moves toward a more positive potential, while the reduction peak potential moves toward a more negative potential. The peak current has a good linear relationship with the scan rate, indicating the electrochemical redox process of capsaicin on the Fe^III^-HMOF-5/SPCE surface is controlled by adsorption.

**FIGURE 7:**
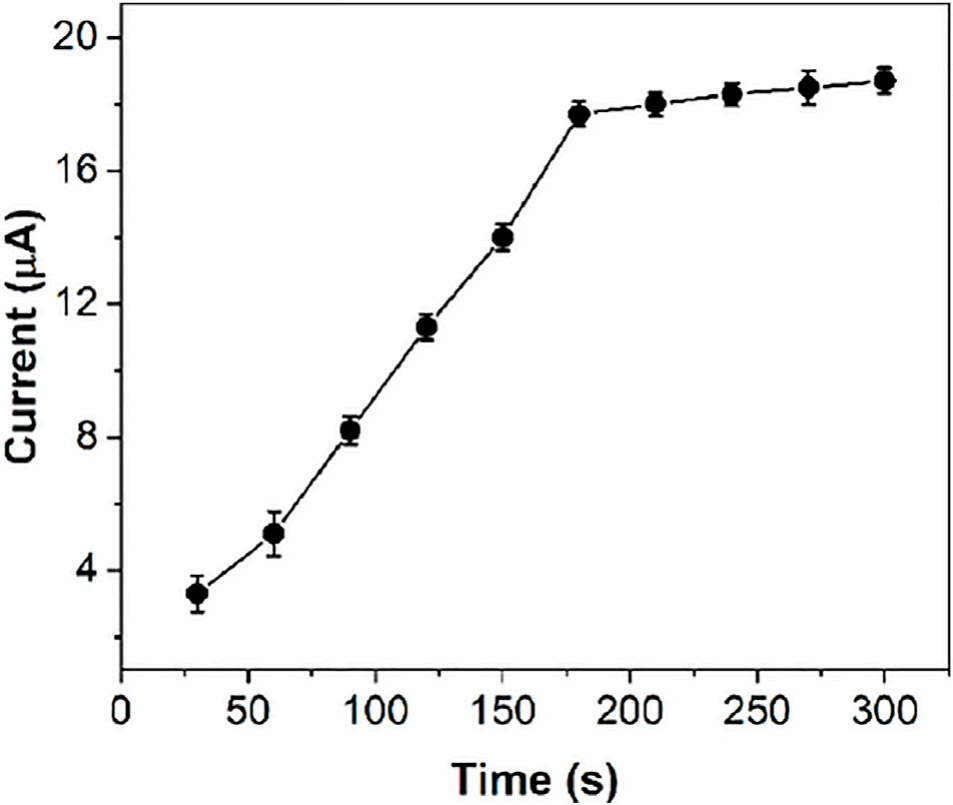
The effect of accumulation time on the anodic peak current of 50 μM capsaicin at Fe^III^-HMOF-5/SPCE.

Since Fe^III^-HMOF-5 can adsorb capsaicin, the accumulation of capsaicin before detection is beneficial to improve the sensitivity of sensing. Figure 7 shows the electrochemical response currents of 50 μM capsaicin under the same conditions at different accumulation times. In the range of 30–180 s, the oxidation peak current of capsaicin on Fe^III^-HMOF-5/SPCE increased significantly with the increase of accumulation time. However, the peak current increase rate decreases after the accumulation time is longer than 180 s. This is because the adsorption and dissolution of capsaicin on the electrode surface tend to reach an equilibrium state. Although the sensitivity of capsaicin detection could be improved by increasing the accumulation time, 300 s was chosen as the optimal deposition time in consideration of the detection efficiency and the detection limit.

We conducted CV scans with Fe^III^-HMOF-5/SPCE in BR buffers containing 50 μM capsaicin at pH 1.5, 3.5, 5.5 and 7.5. The redox peak current and the peak potential of capsaicin were strongly influenced by the pH value. The peak current decreases with increasing pH, which is due to the partial deprotonation of the phenolic structure of the capsaicin molecule. At near-neutral conditions, the CV curves showed almost no redox peaks. In addition, the increase in pH shifted the capsaicin peak potential to a more negative direction of potential. Considering the sensitivity, pH = 1.5 was chosen as the optimal pH condition in this study. In order to study the process of capsaicin redox reaction, the relationship between pH value and oxidation peak potential was investigated. The linear equation was E_pa_(mV) = 610–63.2pH with a slope of 63.2 mV/pH, which is close to the theoretical value of 59.0 mV/pH. According to the Nernst equation, it can be concluded that the electrochemical redox process of capsaicin included equal number of electron and proton.

**FIGURE 8:**
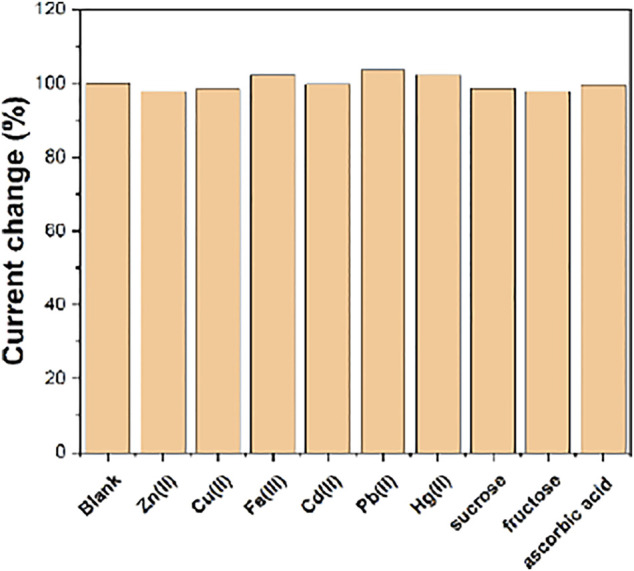
The anti-interference of Fe^III^-HMOF-5/SPCE towards 50 μM capsaicin and 100-folds of interference species.

Figure 8 shows the anti-interference performance of the Fe^III^-HMOF-5/SPCE. It can be seen that the presence of 100-folds of heavy metal ions, sucrose, fructose, ascorbic acid have no effect on the sensing performance.

**FIGURE 9:**
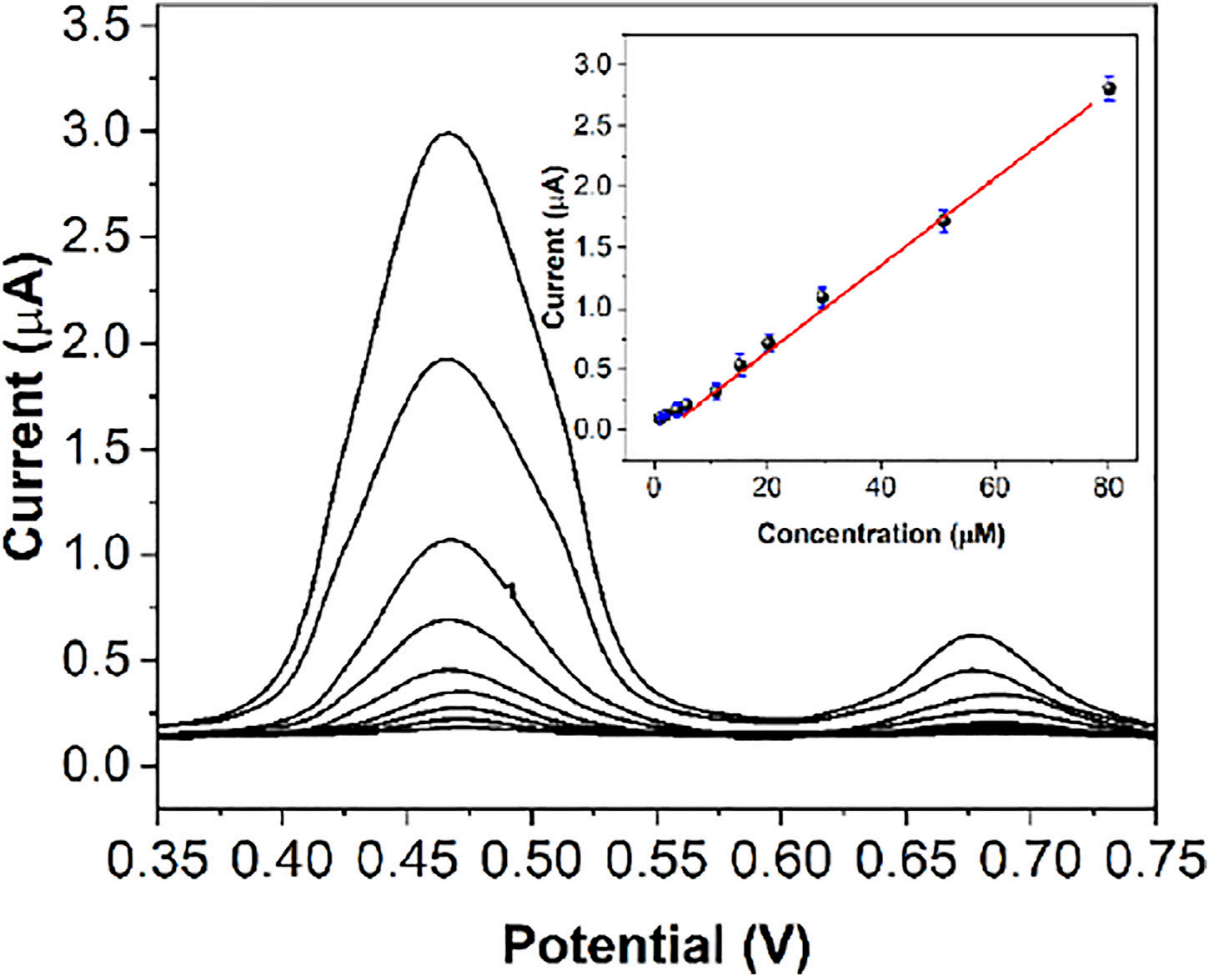
DPV responses of capsaicin at the Fe^III^-HMOF-5/SPCE with different concentrations [**(A–I)**i: 200 nM to 80 μM]. Inset: The calibration plot of capsaicin.

Figure 9 shows the DPV response curves of Fe^III^-HMOF-5/SPCE to successive addition of certain concentrations of capsaicin. As can be seen from the figure, the oxidation peak current increases linearly with increasing concentration of capsaicin. The correlation equation was Ipa (μA) = 0.033 c (μM) + 0.046 (*R* ^2^ = 0.9961) in the range of 200 nM to 80 μM. The limit of detection was calculated to be 68 nM based on signal-to-noise ratio of 3 (Supplementary Figure S3A). The limit of quantification was tested to be 150 nM (Supplementary Figure S3B). Table 1 shows the sensing performance of the proposed electrochemical sensor with other electroanalytical methods. The LOD of the proposed sensor is not the lowest among the recently published articles. However, the proposed sensor has an excellent linear detection range. Since Fe^III^-HMOF-5/SPCE is a portable sensing chip, it exhibits potential field applications.

**TABLE 1:** Linear range and LOD obtained using Fe^III^-HMOF-5/SPCE and other sensors reported for the capsaicin sensing.

Sensor	Linear range	LOD (nM)	References
CeO_2_/SWNT/GCE	100 nM–7.5 μM	28	Ziyatdinova et al. (2020)
RGO/SPCE	200 nM to 22 μM	20	Numphud et al. (2020)
*β*-cyclodextrin/SPCE	1.3–9.3 μM	210	Díaz de León Zavala et al. (2018)
Enz/MWCN/Pt electrode	20–100 μM	610	Sabela et al. (2016)
10% CB−SPE	80 nM to 6 μM	28	Deroco et al. (2020)
Fe^III^-HMOF-5/SPCE	200 nM to 80 μM	68	This work

Six Fe^III^-HMOF-5/SPCE were prepared in the same way and 50 μM capsaicin was measured under the same conditions. The relative standard deviation (RSD) of the results obtained was 4.7%, indicating the proposed sensor exhibited an excellent reproducibility and sensing accuracy. Since the working electrode surface of SPCE is a carbon paste. It tends to adsorb and bind better for nanomaterials than other carbon electrodes (such as glassy carbon) and metal electrodes. Meanwhile, PVP was used in the synthesis of Fe^III^-HMOF-5, which allowed the Fe^III^-HMOF-5to exhibit stable immobilization on the electrode surface. The RSD for the determination of the same concentration of capsaicin at the same electrode after 2 weeks was about 2.6%, suggesting the sensor had an excellent stability.

### Real Sample Test

We tried to apply the prepared Fe^III^-HMOF-5/SPCE electrochemical sensor to the determination of capsaicin content in actual pepper and ICO. A certain volume of extract was added to 10 ml of BR buffer (pH 1.5), and the content of capsaicin in the actual sample was determined by DPV. To evaluate the reliability of the method, UV-vis spectroscopic method was conducted for comparison. The results were statistically evaluated using *t*-test. As shown in Table 2, the results deduced from electrochemical sensor are in good agreement with the UV-vis spectroscopy values.

**TABLE 2:** Determination results and recoveries of capsaicin content in actual pepper and ICO samples using electrochemical sensor (*n* = 5) and UV-vis spectroscopy (*n* = 5).

Sample	Detection (μM)	UV-vis spectroscopy (μM)	*t* Value
Pepper 1	38.07 ± 0.24	38.21 ± 0.22	1.20582
Pepper 2	44.79 ± 0.19	45.07 ± 0.26	2.21776
ICO 1	27.03 ± 0.14	26.90 ± 0.19	1.72949

## Conclusion

In conclusion, this work proposed an electrochemical sensor for capsaicin determination. A bimetallic MOF nanocage (Fe^III^-HMOF-5) was successfully prepared by a one-step solvothermal method and used for SPCE modification. The Fe^III^-HMOF-5 has been characterized to prove its ability of adsorption of capsaicin in its cavities. The Fe^III^-HMOF-5/SPCE shows a superior sensing performance towards capsaicin. It can detect capsaicin linearly in the range between 0.2–80 μM with a low limit of detection of 0.068 μM. In addition, the proposed electrochemical sensor has been used for determination of capsaicin in pepper and ICO samples. The practical performance of the proposed electrochemical sensor was comparable to conventional UV spectrophotometry.

## Data Availability

The original contributions presented in the study are included in the article/Supplementary Material, further inquiries can be directed to the corresponding author.
