# Supplementary material for: Highly Sensitive Capsaicin Electrochemical Sensor Based on Bimetallic Metal-Organic Framework Nanocage
Source: Front Chem. 2022 Feb 15;10:822619. doi: 10.3389/fchem.2022.822619 (PMC8885624; doi:10.3389/fchem.2022.822619)
Supplement: Supplementary file 1 [file DataSheet1.docx]

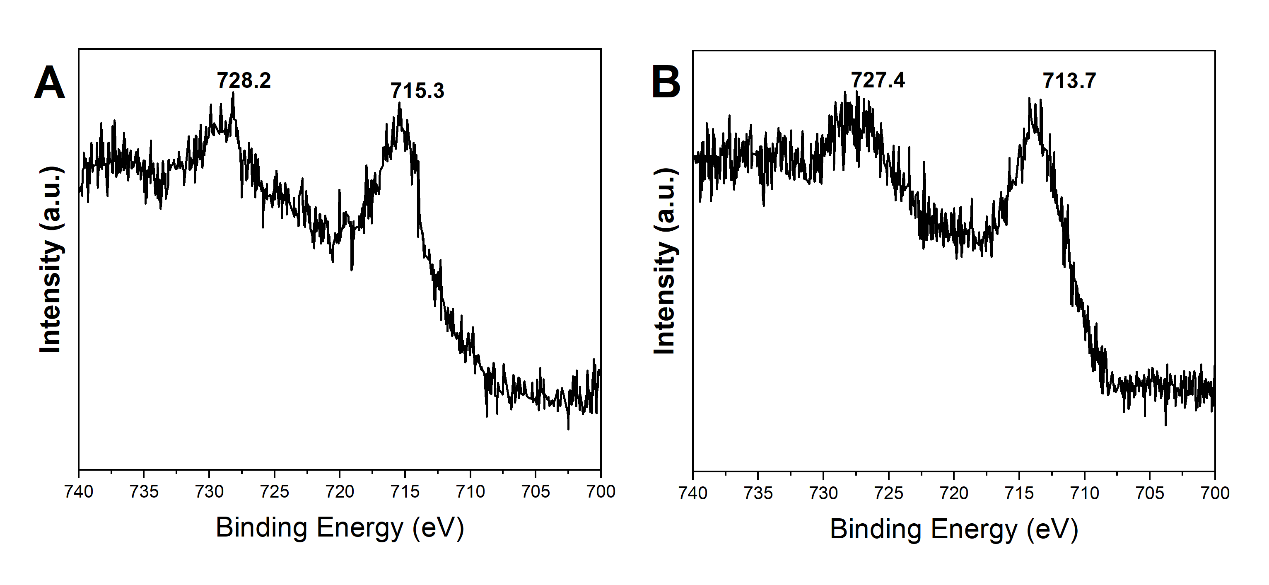


**Figure S1.** Fe2p spectra of (A) Fe^III^-HMOF-5 and (B) Fe^III^-HMOF-5/capsaicin.


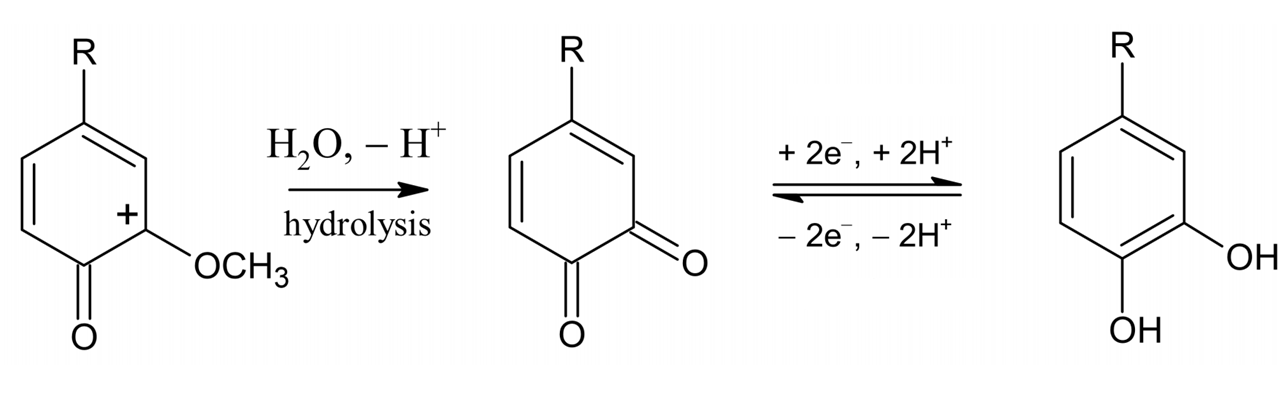


**Figure S2.** Schematic representation of electrochemical oxidation/reduction of capsaicin.


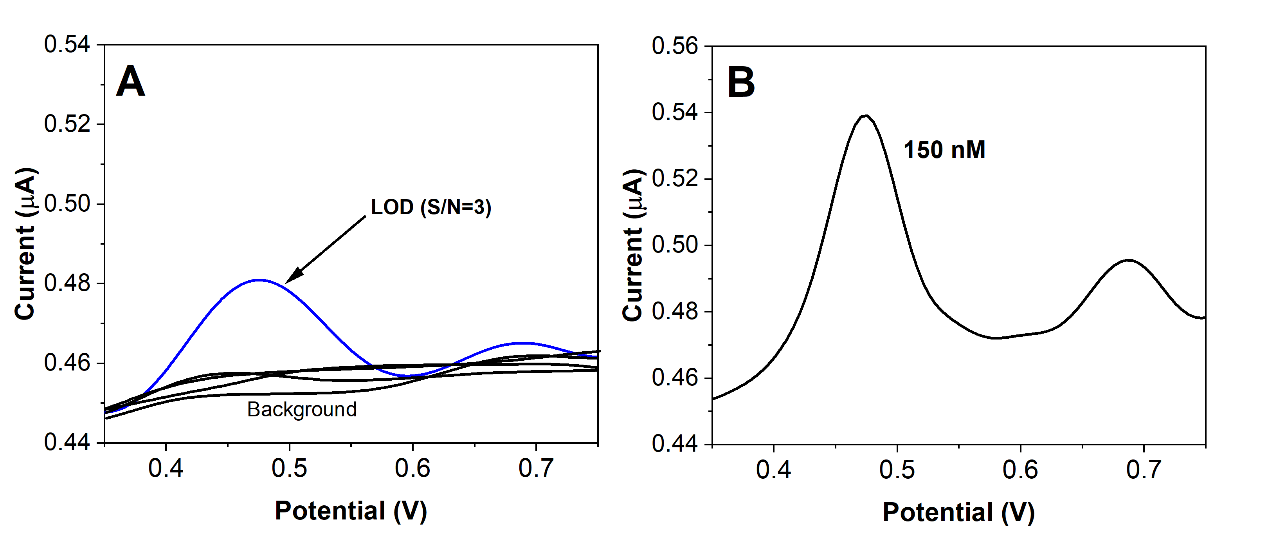


**Figure S3.** DPV responses of (A) LOD and (B) 150 nM of capsaicin.
